# Transcription Factor Ets1 Cooperates with Estrogen Receptor α to Stimulate Estradiol-Dependent Growth in Breast Cancer Cells and Tumors

**DOI:** 10.1371/journal.pone.0068815

**Published:** 2013-07-09

**Authors:** Brian T. Kalet, Sara R. Anglin, Anne Handschy, Liza E. O’Donoghue, Charles Halsey, Laura Chubb, Christopher Korch, Dawn L. Duval

**Affiliations:** 1 Flint Animal Cancer Center, Department of Clinical Sciences, Colorado State University, Fort Collins, Colorado, United States of America; 2 Department of Medicine, University of Colorado School of Medicine, Aurora, Colorado, United States of America; 3 University of Colorado Comprehensive Cancer Center, Aurora, Colorado, United States of America; Institut de Génomique Fonctionnelle de Lyon, France

## Abstract

The purpose of this study was to explore the role of transcription factor Ets1 in estrogen receptor α (ERα)-positive breast cancer progression. We expressed human Ets1 or empty vector in four human ERα-positive breast cancer cell lines and observed increased colony formation. Further examination of cellular responses in stable Ets1-expressing MCF7 clones displayed increased proliferation, migration, and invasion. Ets1-expressing MCF7 tumors grown in the mammary fat pads of nude mice exhibited increased rates of tumor growth (7.36±2.47 mm^3^/day) compared to control MCF7 tumors (2.52±1.70 mm^3^/day), but maintained their dependence on estradiol for tumor growth. Proliferation marker Ki-67 staining was not different between control and Ets1-expressing tumors, but Ets1-expressing tumors exhibited large necrotic centers and elevated apoptotic staining. Ets1 was shown to cooperate with ERα and the p160 nuclear receptor coactivator (NCOA/SRC) family to increase activation of a consensus estrogen response element luciferase reporter construct. Ets1-expressing MCF7 cells also exhibited elevated expression of the ERα target genes, progesterone receptor and trefoil factor 1. Using GST-pulldown assays, Ets1 formed stable complexes containing both ERα and p160 nuclear receptor coactivators. Taken together, these data suggest that the Ets1-dependent estradiol sensitization of breast cancer cells and tumors may be partially due to the ability of Ets1 to cooperate with ERα and nuclear receptor coactivators to stimulate transcriptional activity of estrogen-dependent genes.

## Introduction

Approximately 75% of breast cancers are estrogen and progesterone receptor (PGR)-positive and treatments to block the mitogenic activity of estrogen are a standard therapy [1], [2]. Unfortunately, 30–50% of estrogen receptor (ER)-positive breast cancers fail to benefit from or acquire resistance to these therapies [3]. Elevated expression of Ets transcription factors including Ets1, Ets2, ER81, PEA3 and ESE1 have been identified in epithelial cancers (breast, lung, prostate, colon) [4] and are expressed at higher levels in metastatic lesions thus serving as independent predictors of poor prognosis [5]–[11]. This phenotype has been associated with Ets regulation of HER2 epidermal growth factor receptor, matrix metalloproteinases, heparinase, VEGF and other genes involved in growth, tissue remodeling, and angiogenesis [8]. Both Ets1 and Ets2 expression are correlated with decreased disease-free survival in breast cancer patients and a dominant negative Ets construct can inhibit the anchorage independent growth of breast cancer cell lines [12], [13]. In addition, elevated expression of Ets1 and Ets2 identified in invasive breast cancers has been correlated with increased expression of the p160 nuclear receptor coactivators NCOA1 (SRC1) and NCOA3 (AIB/SRC3) [5]. Deep sequencing of mRNA isolated from a panel of breast cancer cell lines found that direct comparison of transcripts from ER positive lines and ER negative lines identified a 922-fold elevation in *ETS1* expression in the ER negative lines [14]. In fact, RT-qPCR analysis of multiple Ets factors in human breast cell lines showed that *ETS1* expression was undetectable in ERα positive breast cancer lines, but expressed in ERα negative lines [15]. However, a recent DNA methylation study has revealed that some ER positive breast tumors exhibit *ETS1* demethylation, suggesting that a subset of ER positive tumors express elevated levels of *ETS1* [16].

The Ets family of transcription factors includes approximately 30 members that bind to a consensus 5′-GGA(A/T)-3′ binding site through a highly-conserved 85 amino acid Ets DNA-binding domain (DBD) [17], [18]. Many of the Ets transcription factors are activated in response to receptor tyrosine kinase activation of the Ras/Raf/MAP kinase pathway [19]. MAP kinase phosphorylation of a conserved domain in the amino terminus of Ets1/2 results in recruitment of the CBP/p300 coactivators and transactivation [20]. In addition, Ets factors, including Ets1, Ets2, ETV4/PEA3 and ER81, physically interact with members of the nuclear receptor coactivator (NCOA) family [5], [21], [22]. The p160 nuclear receptor coactivator family consists of three primary homologous family members NCOA1, NCOA2, and NCOA3 (see [23] for review). A review of the role of NCOA members in cancer indicate that NCOA1 and 3 are associated with breast cancer initiation and progression [24]. Reducing expression of these NCOA factors results in decreased ERα dependent growth and elevated expression is often correlated with reduced disease free survival. The NCOA family directs the formation of a transcriptional complex with histone acetylase transferases (CREB binding protein (CBP)/p300, p/CAF) and methyl transferases (CARM-1, and PRMT1). In addition to nuclear hormone receptors, NCOA binding and transcriptional activation has also been identified with other transcription factors including AP-1, NFκB, p53, STATs, and Ets factors [5], [22], [23], [25]. ER81 binding to NCOA3 appears to occur through binding of the Ets DBD and a flanking amino-terminal inhibitory domain to the amino-terminal 810 amino acids of NCOA3 [22]. This region of NCOA3 contains a conserved domain implicated in DNA binding and protein dimerization as well as the receptor interaction domain (RID) which contains three conserved LXXLL motifs that bind to the activation function-2 (AF-2) domain of nuclear hormone receptors. Ets1 has also been shown to directly interact with several members of the nuclear hormone receptor family including the vitamin D receptor, ERα, androgen receptor and the peroxisome proliferator-activated receptor-α (PPARα) to induce ligand independent transcriptional activation [26], [27]. In addition, the Ets factor, GA-binding protein β has been shown to interact with unliganded glucocorticoid receptor to stimulate *BRCA1* [28]. Structure function analysis indicates that the Ets1 DBD and adjacent amino-terminal inhibitory domain appear to interact with the A/B/C domains of the vitamin D and PPARα receptors [26]. Thus, the binding of Ets1 to ERα appears to augment transcriptional activation by serving as a bridging molecule to attract NCOA complexes [26]. Based on these interactions, we hypothesized that Ets1 could serve as an initiating switch reducing the dependence of breast cancer cell lines on ERα signaling and eventually leading to hormone independence.

In the current study, we examine the effect of Ets1 expression on human breast cancer cell lines using both *in vitro* and *in vivo* analyses. Our studies show that Ets1 expression increases cellular proliferation, migration, and invasion. In addition, *in vivo* Ets1 expression increases 17β-estradiol-dependent tumor growth. Functional studies of transcriptional activation suggest that these changes may be due to increased ERα signaling from Ets1 complexes containing both ERα and members of the p160 family of nuclear receptor coactivators.

## Materials and Methods

### Cell Culture, Generation of Stable Cell Lines and Western Blotting

BT-474, MCF10A, MDA-MB-157, MDA-MB-231, MDA-MB-453, T-47D and ZR-75-1 cells were purchased from the University of Colorado Cancer Center Tissue Culture Shared Resource as short tandem repeat analysis validated stocks [29]–[34]. HeLa cells were a gift from Arthur Gutierrez-Hartmann, and were validated by short tandem repeat analysis. MCF7 cells were purchased from the American Type Culture Collection. BT-474, MDA-MB-157, MDA-MB-231, MDA-MB-453, T-47D and ZR-75-1 were cultured in RPMI 1640 supplemented with 1 mM sodium pyruvate, 20 mM HEPES, 4 mM L-glutamine and 10% (v/v) fetal bovine serum (FBS; Atlas Biologicals). HeLa cells were cultured in Dulbecco’s Modified Eagles Medium (DMEM) high glucose supplemented with 1X non-essential amino acids, 10 mM HEPES, 2 mM L-glutamine, 2.5% (v/v) FBS and 15% donor horse serum (Mediatech). MCF7 cells were cultured in DMEM high glucose supplemented with 10% (v/v) FBS, 10 mM HEPES, 1 mM sodium pyruvate, 1X non-essential amino acids and except where indicated 0.001% Humulin™. MCF10A cells were cultured in DME/F-12 1:1, supplemented with 20 ng/mL epidermal growth factor, 0.5 µg/mL hydrocortisone, 0.0135 U/mL Novolin, 0.1 µg/mL cholera toxin and 5% FBS. Cells were electroporated (10^6^ cells, 140 V, 70 msec, 1 pulse) with 10 µg of pCTV or pCTV-HAhEts1 plasmid DNA and selected with 500 µg/mL G418 sulfate. Ets1-expressing clonal isolates and the pCTV clonal pool were electroporated with 10 µg RSVpGL4.17 and 0.5 µg pCDNA-hygromycin (used as a selection marker) and selected in hygromycin B sulfate. Clonal isolates were screened for HA-Ets1 expression by immunoprecipitation (HA.11 antibody, Covance) and Western Blot analysis using an Ets1 antibody (N-276, Santa Cruz Biotechnology). Cell lysates were assessed by Western Blot analysis for ERα (1∶1000, Santa Cruz Biotechnology) and tubulin loading control (1∶10,000, Sigma) using chemiluminescent imaging. Luciferase expression was assessed by bioluminescent imaging with 50 µM luciferin (Xenogen: LivingImage Software, 1 min, medium binning, f-stop 1). Antibiotic selection markers were omitted for two weeks to verify stability of Ets1 and luciferase expression. At study completion, MCF7 clonal isolates were validated by short tandem repeat analysis (University of Colorado Cancer Center DNA Sequencing Shared Resource).

### RNA Isolation, cDNA Synthesis and Quantitative Real-time PCR

RNA was isolated from cells using the RNeasy Mini Kit (Qiagen). cDNA was synthesized using the QuantiTect Reverse Transcription Kit (Qiagen). Quantitative real-time PCR was performed with denaturation at 95°C for 10 minutes and 40 cycles of 30 seconds at 95°C and 60 seconds at 60°C using the iQ SYBR Green Supermix (BioRad) on the Stratagene Mx3000P instrument. Primers were designed to be intron-spanning using Primer-BLAST. The standard curves, dissociation curves and amplification data were collected using Mx3000P software and analyzed with the 2^(−ΔΔCt)^ method [35]. Expression levels were normalized to hypoxanthine phosphoribosyltransferase 1 (*HPRT1*) expression. Primer sequences are shown in Table S1.

### Plasmid Construction

The coding sequence for human *ETS1* (provided by Bo Wasylyk, IGBMC, Illkirch, France) was PCR amplified from pSG5-hEts1 [36] using primers that incorporate an influenza hemagglutinin epitope (HA) – tag at the amino-terminus: hEts1 S 5′-GAA GAT CTC CAT GGC TTA TCC TTA TGA CGT GCC TGA CTA TGC CTC AAG CTT AAA GGC GGC CGT CGA TC-3′, hEts1 AS 5′-ACG CGT CGA CTG CCA TCA CTC GTC GG -3′. The amplified *HA-hETS1* cDNA was subcloned into pEGFP-C1 from which the EGFP coding sequence was removed (pCTV) and verified by sequencing. RSVpGL4.17 was constructed by subcloning a *Hind*-III fragment containing the Rous Sarcoma Virus 5′ LTR (RSV) promoter into pGL4.17. PCR3.1SRC1/NCOA1, SRC2/NCOA2 and SRC3/NCOA3 were provided by Drs. Bert O’Malley and Carolyn Smith, Baylor College of Medicine.

### Clonogenic Assay

Transiently transfected MCF7, BT-474, T-47D and ZR-75-1 cells were plated at a density of approximately 1 cell/mm^2^ in either supplemented DMEM media with 1% FBS (MCF7) or supplemented RPMI 1640 media with 10% FBS (BT-474, T-47D and ZR-75-1). Stable MCF7 clones were plated at a density of approximately 1 cell/mm^2^ in phenol red/insulin free media and treated with 10 nM 17β-estradiol or vehicle. Media was changed at least twice per week. After 14 days, cells were fixed with methanol, stained with 0.3% crystal violet and colonies were counted.

### Cell Proliferation and Growth Inhibition Assays

MCF7 cells were plated into 96-well plates (2,000 cells/well) in media without insulin and treated with 4-hydroxytamoxifen. Cell numbers were assessed using a resazurin-based bioreductive fluorometric assay [37]. IC_50_ values were calculated by fitting to a sigmoidal dose-response curve using GraphPad Prism 5.

### Invasion and Migration Assays

Cells were resuspended in media, free of serum, phenol red and insulin, and plated at 50,000 cells/well in 24-well Matrigel Invasion Chamber plates (BD BioSciences) using 5% FBS as chemoattractant. After 24 hours, migratory and invasive cells were fixed, stained and counted.

### Experimental Animals and MCF7 Orthotopic Tumor Model

Animal procedures were approved by the Colorado State University IACUC committee (Protocol # 08-261A-01). Female athymic nu/nu mice (5–6 weeks) were obtained from NCI-Frederick and housed 5 mice/isolator cage. After one week of acclimation the mice were ovariectomized and subcutaneously implanted with silastic pellets containing either cellulose/17β-estradiol mixture (2 mg/pellet) or cellulose alone (n = 20/group) [38]. After one week, 2×10^6^ cells (100 µL of 50% Matrigel) were injected into the right mammary fat pad (n = 10/group). Tumor volume was calculated using the 3.14 × length(w^2^)/6 using caliper measurements. After one week and at study completion, mice were injected with luciferin (126 mg/kg i.p.) and tumors were assessed using bioluminescent imaging (Xenogen, LivingImage software). At study completion, tumor volumes and weight of excised tumors and uteri were obtained.

### Immunohistochemistry of Tumors

Formalin-fixed, paraffin embedded tumor sections (5 µm) were deparaffinized and rehydrated in xylene and graded ethanol baths. Sections were treated at 125°C for 1 min in Citra Antigen retrieval solution pH 6.0, blocked with Background Sniper (Biocare Medical) blocking reagent for 10 minutes at room temperature and incubated with Ki-67 antibody at 4°C overnight, followed by a Texas Red-labeled anti-rabbit secondary antibody for 45 minutes at room temperature. TUNEL staining was performed according to manufacturer’s instructions (12156792910, Roche). For CD31 and Ki-67 dual staining, sections were incubated with anti-mouse CD31/PECAM (Novus Biologicals; rat monoclonal, 1∶50, 90 min at RT) and anti-human Ki-67 (Epitomics; rabbit monoclonal, 1∶100, 60 min at RT). Slides were then incubated with secondary antibodies fluorescein goat anti-rabbit (Vector Laboratories; 1∶100, 60 min at RT) and Alexa Fluor 594 chicken anti-rat (Invitrogen; 1∶100, 60 min at RT). Image analysis was performed using AxioVision 4.3 system software from Carl Zeiss.

### Apoptosis Assay

Apoptosis was measured as previously described [39] with minor modifications. Cells were plated at a density of 500,000 cells per well of a 6 well plate. The following day, wells were washed with 3 mL DPBS and 3 mL of DMEM containing no FBS was added. After 24 hours, media was collected, cells were washed with DPBS and wash was collected, then cells were trypsinized. Trypsinized cells, aspirated media and wash DPBS for each sample were pooled and centrifuged for 5 min at 500 x g at 4°C. Cells were washed twice with 5 mL cold DPBS, then stained using the FITC Annexin V Apoptosis Detection Kit (556547, BD Biosciences). Flow cytometric analyses were performed using a CyAn ADP flow cytometer (Beckman-Coulter).

### GST Pulldown Assays

Recombinant fusion proteins were prepared using glutathione-sepharose resin as previously described [40]. Fluorotect Green_Lys_-labeled proteins were synthesized from 1 µg pcDNA3.1 ERα or PCR3.1 NCOAs using the TNT coupled transcription-translation reticulocyte lysate system with T7 polymerase (50 µL reactions). Control and GST-Ets1 fusion proteins (4 µg/20 µL resin) in 0.5 mL binding buffer (40 mM HEPES, pH 7.5, 100 mM NaCl, 5 mM MgCl_2_, 0.1 mM EDTA, 0.05% Nonidet P-40, 1 mM dithiothreitol, and 1X complete protease inhibitors (Roche) were incubated with Green_Lys_-labeled ERα (5 µL) for 30 minutes at 4°C. Where indicated, Green_Lys_–labeled NCOA proteins (5 µL) were added and incubated for an additional 2 hours. The resin was pelleted (1000 × g for 2 minutes) and washed 3 times with 1 mL of binding buffer with 0.1% Triton X-100. Bound Green_Lys_ -labeled proteins were eluted in SDS sample buffer (65°C for 10 min), analyzed by SDS-PAGE and quantified with a Typhoon 9410 imager (488 nm laser, 650 volts, 520BP40 filter) with Imagequant software.

### HeLa Transient Transfection

HeLa cells (20,000 cells/well of a 96-well plate) were transiently transfected with 75 ng of a luciferase reporter with two copies of a consensus estrogen responsive element linked to a thymidine kinase minimal promoter (ERE2-TK-LUC), 0.2 ng pcDNA3.1-hERα and 50 ng pSG5-hEts1 using Effectene transfection reagent in Optimem serum free medium. Hrl-TK-renilla luciferase plasmid (1 ng) was included to control for transfection efficiency and DNA concentrations were held constant with control plasmids. Treatment in fresh media was initiated the next day with 10 nM 17β-estradiol where indicated. After 24 hours, cells were harvested and assayed for luciferase and renilla luciferase activity using Stop ‘N Glo reagents on a BioTek Clarity plate-reading luminometer. Firefly luciferase activity in each sample was normalized for Renilla luciferase activity.

## Results

### Ets1 Expression Increases Cellular Proliferation, Colony Formation, Migration and Invasion of Human Breast Cancer Cells *in vitro*

**Figure 1:**
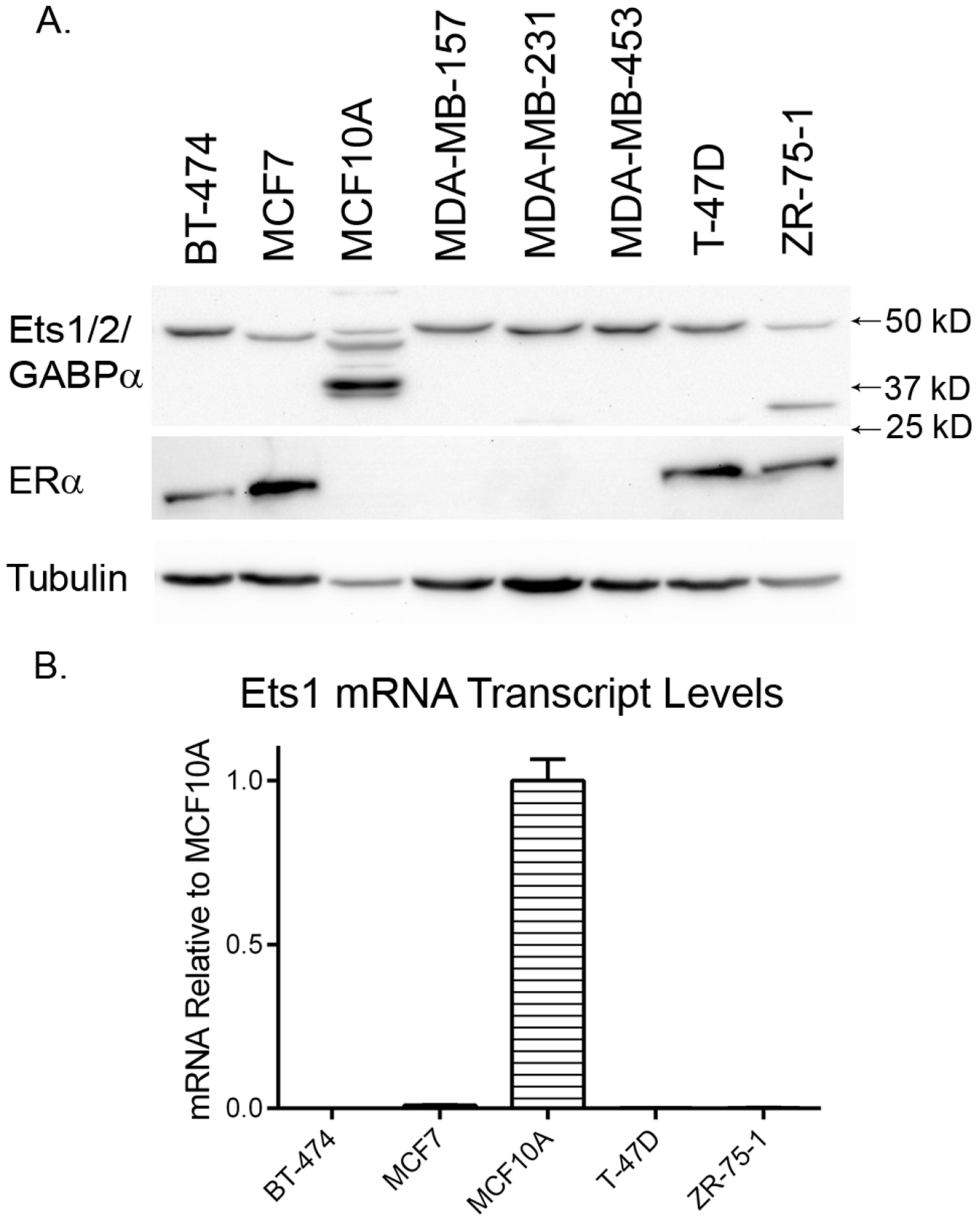
Ets1 and ERα expression in human breast cell lines. A. Breast cell lines were analyzed by Western blot for ERα and Ets1 with tubulin as a loading control. B. ER-positive BT-474, MCF7, T47D and ZR-75-1 cells were analyzed by qRT-PCR for Ets1 transcript levels. Data represent mean ± standard deviation. Ets1 transcript levels are statistically significantly lower in BT-474, MCF7, T-47D and ZR-75-1 cells compared to MCF10A cells by ANOVA with Tukey’s post-test analysis p<0.001.

To determine if Ets1 expression was sufficient to alter the growth characteristics of ER-positive breast cancer cell lines, we evaluated a panel of breast cancer cell lines including BT-474, MCF7, MDA-MB-157, MDA-MB-231, MDA-MB-453, T-47D and ZR-75-1 as well as the human non-tumorigenic mammary cell line MCF10A for expression of ERα and Ets1. Western blot analysis confirmed that only four of the eight human breast cell lines expressed ERα: BT-474, MCF7, T-47D and ZR-75-1 (Figure 1A) [41]. Western blot analysis using an antibody targeting the amino-terminus of Ets1(N-276), detected bands in each of the cell lines at approximately 50 kD, a band at approximately 27 kD in the ZR-75-1 line and a cluster of bands located between approximately 30 and 75 kD in the MCF10A line. This result was surprising since previous studies have shown limited Ets1 expression in many of these cell lines [10], [15]. However, BLAST analysis of the epitope for this antibody (amino acids 55–70 [42]) reveals that this epitope is 86 and 82% conserved with the corresponding regions in the Ets factors, Ets2 and GA binding protein α, respectively. This suggests that the antibody is likely to exhibit affinity for these three Ets transcription factors and their predicted splice isoforms, which range in size from approximately 67 to 20 kD. Consequently, we measured *ETS1* expression in the four ERα positive breast cell lines relative to the non-tumorigenic human breast cell line MCF10A by quantitative real-time PCR (Figure 1B). All four cell lines displayed over 500-fold lower *ETS1* expression than MCF10A. These studies confirmed the lack of *ETS1* expression previously described in these ERα-positive breast cancer lines [15].

Clonogenic assays were performed with the BT-474, MCF7, T-47D and ZR-75-1 ER-positive, Ets1-negative human breast cancer cells after transient transfection with Ets1 or empty vector (Table 1). Ets1 expression increased colony formation in MCF7 (2-fold), T-47D (2-fold) and ZR-75-1 (6-fold).

**Table 1:** Colonies formed as a function of Ets1 expression.

Cell Line	Ets1	Empty Vector
BT-474	93±7	89±15
MCF7	455±64^a^	242±24
T-47D	295±7^b^	149±4
ZR-75-1	27±4^c^	5±4

a p = 0.047 compared to MCF7 transfected with empty vector.

b p<0.0001 compared to T-47D transfected with empty vector.

c p = 0.0018 compared to ZR-75-1 transfected with empty vector.

**Figure 2:**
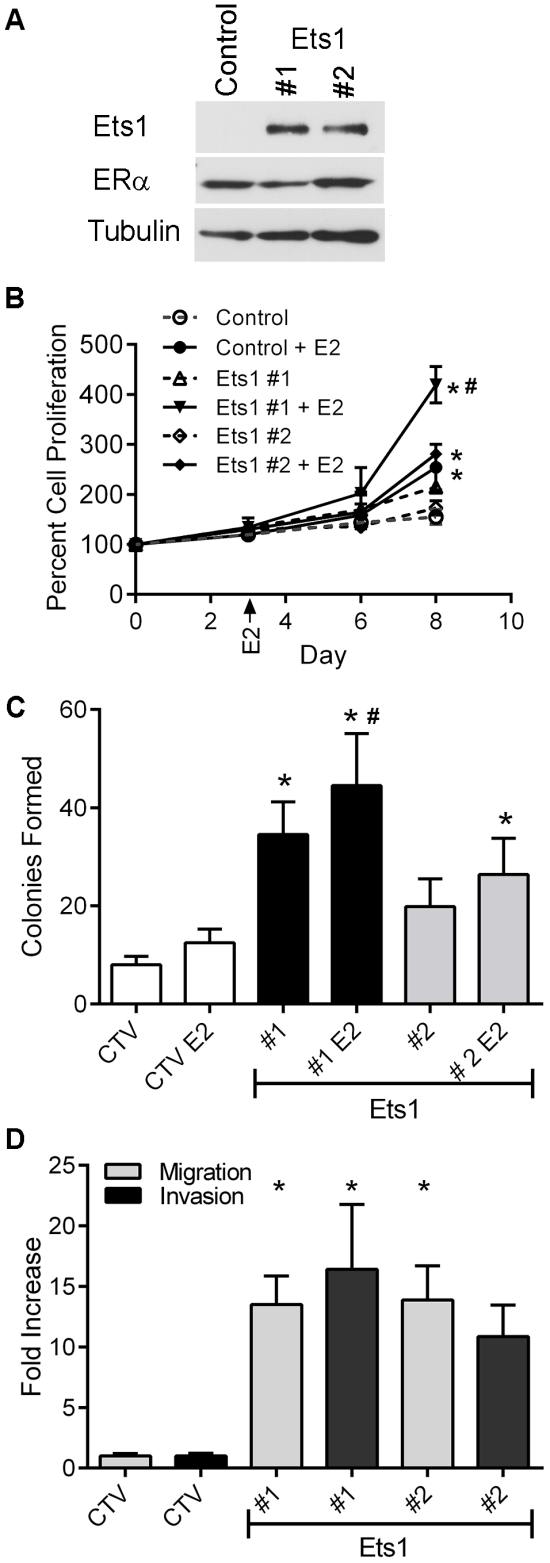
Ets1 expression stimulates cellular proliferation, colony formation and migration in MCF7 cells. A. Whole cell lysates from MCF7 control and Ets1 stable transformants with equal amounts of total protein were immunoprecipitated with an anti-HA.11 antibody and analyzed by Western blot for Ets1expression. Corresponding whole cell lysates were assessed by Western blot analysis for ERα with anti-tubulin as a loading control. B. Stable transformants were plated at a density of 2000 cells/well in phenol red free media with 5% charcoal dextran stripped serum. On Day 3, the wells were treated with either 0.1% ethanol vehicle, or 10 nM 17β-estradiol (E2). Cell growth was assessed over eight days using a resazurin-based assay. Data represent the mean ± SEM of two independent experiments. * Different from control or non-estradiol stimulated culture by ANOVA with Tukey’s post-test analysis p<0.05. # Different from control+E2 p<0.05. C. Control and Ets1-expressing stable transformants were plated at a density of 800 cells/well. Estradiol (10 nM) was added as indicated. After 13–14 days colonies containing ≥50 cells were counted. Data represent the mean ± SEM of five independent experiments. Significantly different from control (CTV, *) or control+estradiol (#) by ANOVA with Dunnett’s post test analysis p<0.05. D. Log phase control (CTV) and Ets1 stable transformants were resuspended (50,000 cells/well) in serum and phenol red free medium in 24-well BD migration and matrigel invasion chambers with 5% FBS as the chemoattractant. After 24 hours, the filters were scrubbed, fixed, stained and counted. Data represent the mean ± SEM of three independent experiments performed in triplicate. * Significantly different from control by ANOVA with Dunnett’s post test analysis, p<0.05.

Because MCF7 cells are a well characterized, stable epithelioid breast cancer cell line expressing estrogen (Figure 1A), androgen, progesterone and glucocorticoid receptors [30], but not Ets1 (Figure 1B) [10], [41] and display increased clonogenicity upon Ets1 expression (Table 1), we selected them for further analysis. Authenticated MCF7 cells stably expressing HA-epitope tagged human Ets1 and firefly luciferase were developed and Ets1 expression was verified in HA-immunoprecipitated proteins by Western blot utilizing an Ets1 antibody (Figure 2A). ERα expression of the stable clones was not altered as verified by Western blot analysis (Figure 2A).

To validate our stable Ets1-expressing MCF7 model, we measured the estradiol dependence and growth rates of the Ets1-expressing cells compared to control MCF7 cells transfected and selected for the empty vector. Cells were plated in media containing charcoal-dextran stripped FBS. Where indicated, 10 nM 17β-estradiol was added on day three and cell number was assessed periodically for eight days (Figure 2B). In the absence of exogenous 17β-estradiol the cultures exhibited minimal growth and Ets1-expressing clones were not significantly different from the control MCF7 cells. However the addition of estradiol stimulated growth of both control and Ets1-expressing clones. At day eight, control cultures containing estradiol displayed significantly higher cell numbers compared to unstimulated control cultures. Also, the Ets1 expressing clones exhibited significantly more proliferation than the untreated control and their corresponding non-estradiol stimulated cultures (p<0.05). In addition, with estradiol stimulation, average percent cell proliferation values were higher in the Ets1-expressing clones than the control MCF7 cells and by day eight, Ets1 #1 was significantly elevated (p<0.001) (Figure 2B). To further validate our stable Ets1-expressing MCF7 model, we next conducted clonogenic assays in the presence and absence of estradiol. Ets1-expressing clone #1 exhibited a 4-fold increase in colonies formed compared to control (Figure 2C). Clone #2 also had an approximate 2-fold increase compared to control cells, however this change was not statistically significant. Ets1-expressing clones also had larger colonies compared to control MCF7 cells. While estradiol increased colony numbers in both control and Ets1-expressing clones, these increases were not significantly different from untreated cells.

Ets1 transcriptionally promotes proteases involved in extracellular matrix degradation, a key component of tumor invasion [43]. Thus, we investigated the migratory and invasive phenotypes in the Ets1-expressing clones. Ets1-expressing clones exhibited a greater than 10-fold increase in migrating cells (Figure 2D). Similarly, the invasive cells were also increased greater than 10-fold in the Ets1 clones compared to control (Figure 2D).

To determine if Ets1 expression altered the sensitivity of the MCF7 cells to 4-hydroxytamoxifen, IC_50_ values were determined from dose-response curves of growth inhibition for each cell line in response to treatment with 4-hydroxytamoxifen (Table S2). No significant differences in 4-hydroxytamoxifen sensitivity were observed between the Ets1-expressing and control cell lines.

### Ets1 Increases the Growth of Tumors in the Presence of Estradiol

Ets1-expressing human breast cancer cells demonstrated increased cellular proliferation, clonogenic growth, migration and invasion. To determine if Ets1 expression also altered MCF7 growth characteristics *in vivo,* we assessed estrogen-dependent and -independent orthotopic tumor growth. Female athymic nu/nu mice were ovariectomized and subcutaneously implanted with silastic pellets containing either 17β-estradiol or cellulose. These pellets produce serum estradiol levels in mice similar to those of commercially available 60 day slow-release estradiol pellets for at least 6–8 weeks following implantation [44]. At study termination, cellulose pellet bearing mice had average uterine weights of 34.23±12.45 g (control MCF7) and 29.89±9.57 g (Ets1 clone #1), while the estradiol pellet bearing mice had significantly larger uteri with average weights of 216.7±26.02 g and 266.2±75.38 g respectively (p<0.0001). Thus, the pellets released uterine stimulatory levels of 17β-estradiol through the duration of the experiment.

**Figure 3:**
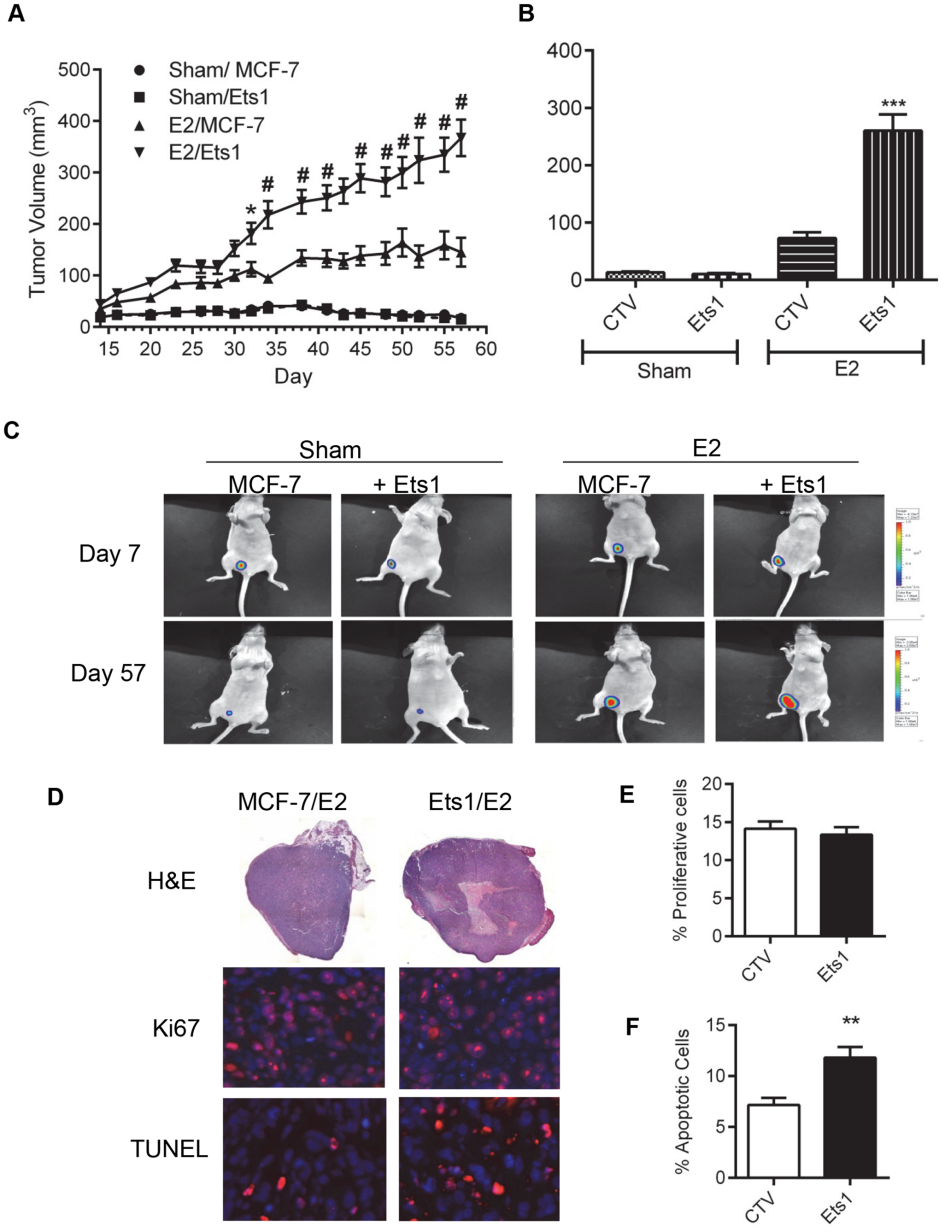
Ets1 increases the growth of tumors in the presence of estradiol. Female athymic nu/nu mice were ovariectomized and subcutaneously implanted with either an estradiol or control silastic pellet. After one week recovery, 2×10^6^ cells of either control or Ets1-expressing stable cells (clonal isolate #1) were injected into the right mammary fat pad (n = 10/group). A. Tumor volume was calculated using the 3.14 x length(w^2^)/6. Values are mean ± SEM. Symbols indicate values that differ significantly from estradiol/MCF7 tumors by ANOVA with Bonferroni’s post-test analysis. * p<0.01, # p<0.001 day 34 to end. B. At end of study, tumors were excised and weighed. *** Significantly different from MCF7/estradiol p<0.001. C. Mice were imaged with the Xenogen Bioluminescent system after one week to assess tumor implantation and at the end of the study. Representative images for each group at constant signal intensity are shown. D. Representative images of hematoxylin and eosin stained tumor sections, Ki67(red), TUNEL (red) overlayed on DAPI (blue) staining in tumors. E. Ki67 stained sections from representative tumors (n = 7). Bars represent mean ± SEM of the average tumor staining. F. TUNEL stained sections from representative tumor sections (n = 7). Bars represent mean ± SEM of the average tumor staining. ** Significantly different by t-test p<0.05.

One week post pellet implantation, luciferase-expressing MCF7 cells were injected into the mammary fat pad of the mice. After one week of tumor growth, luciferase-expressing tumor cells were observed in 100% of the animals by bioluminescent imaging (Figure 3C). The average tumor volume for each group was plotted over eight weeks (Figure 3A). In the absence of estradiol, both the control and Ets1-expressing tumors exhibited minimal growth with no difference in growth rate. As anticipated, due to the established estrogen-dependence of MCF7 cells [45], mice implanted with estradiol-releasing pellets exhibited increased tumor growth for both control and Ets1-expressing MCF7 cells. The rate of growth of the Ets1-expressing tumors (7.36±2.47 mm^3^/day) was significantly higher (p<0.01) than the rate of growth of the control tumors (2.52±1.70 mm^3^/day). Ets1-expressing/estradiol treated tumors showed significantly increased tumor volumes compared to control/cellulose tumors beginning 32 days post tumor implantation and were significantly different from control/estradiol tumors from 34 days post implantation. When the study was terminated at eight weeks, mean tumor volume of Ets1/estradiol tumors was more than twice that of control/estradiol tumors.

Tumor weights at study termination replicated tumor volume measurements (Figure 3B) with estradiol pellet bearing mice having heavier tumors compared to cellulose pellet mice (p<0.001). Ets1/estradiol tumors also had significantly heavier tumors when compared to control/estradiol tumors. These data suggest that Ets1-expression in MCF7 tumors increases growth rate without releasing them from estradiol-dependence.

At study termination, bioluminescent imaging was used to visualize the tumors and identify metastatic lesions (Figure 3C). Mice were initially imaged with the tumor in place, and again after the tumors were excised to detect any signal masked by the high signal intensity of the tumors, but no metastases were detected in any of the mice.

### Immunohistochemical Analysis of TUNEL, Ki67, and CD31 in Ets1-expressing and Control MCF7 Tumors

To better understand the observed difference in tumor growth between the control/estradiol and Ets1/estradiol tumors, immunohistochemical staining for proliferation and apoptosis were performed (Figure 3D). Ki-67 is a nuclear antigen associated with cellular proliferation and is expressed during late G_1_, S, G_2_ and mitosis, but not in resting cells. Both the control and Ets1-expressing tumors showed similar numbers of Ki-67 positive cells, with an average of approximately 14% of the cells in each field exhibiting positive staining (Figure 3E).

Cellular apoptosis was assessed in serial tumor sections using immunohistochemical detection of DNA strand breaks (TUNEL technology). The percentage of apoptotic cells was significantly higher in Ets1 tumors (12%) compared to control tumors (7%, Figures 3D & F). One complication with interpreting these data is that 86% of Ets1/estradiol tumor sections showed central areas of necrosis, while only 29% of controls had necrotic areas (Figure 3D, H&E). Since these necrotic areas exhibit positive staining in the TUNEL assay, finding image fields that met the criteria of being non-necrotic and away from the edge of the tumor was difficult in the Ets1/estradiol samples. In addition, *in vitro* analysis of serum-starved cells showed no difference in annexin V staining between control MCF7 cells and the Ets1 clone #1 cells (Figure S1). Consequently, the increased percentage of apoptotic cells within these tumors may be due to adjacent necrotic regions. Finally, dual CD31 and Ki-67 staining of tumor sections was performed to measure angiogenesis. No significant difference in angiogenesis was found between the Ets1 tumors and control MCF7 tumors removed from estradiol-treated mice (Figure S2).

### Ets1 Cooperates with ERα and p160 Nuclear Receptor Coactivators to Induce Activation of a Consensus Estrogen Response Element

**Figure 4:**
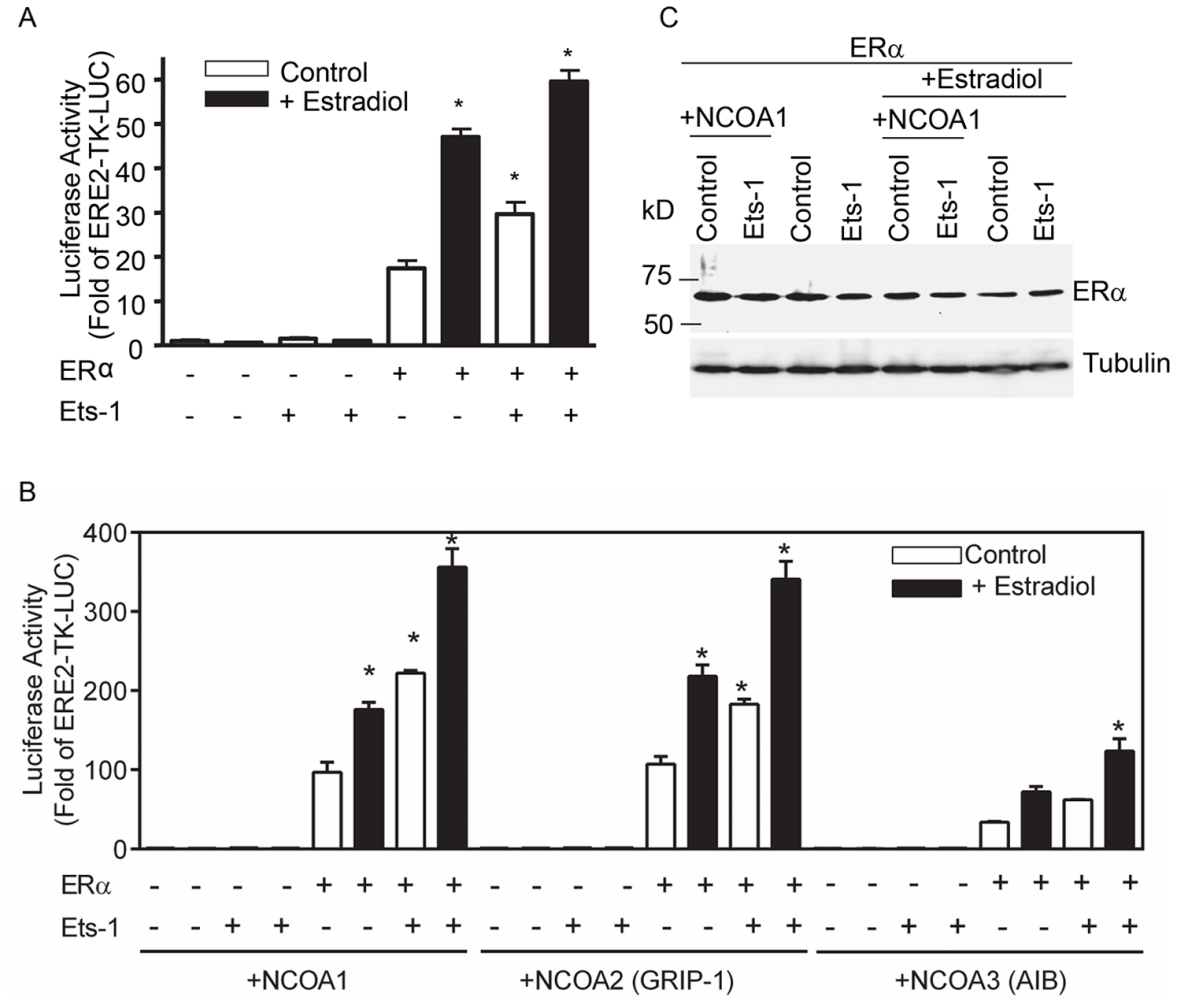
Ets1 cooperates with ERα and nuclear receptor coactivators to stimulate an estrogen responsive promoter. A. HeLa cells were transiently transfected with 75 ng ERE2-TK-LUC, 0.2 ng pcDNA3.1-hERα and 50 ng pSG5-hEts1 or control vector to hold DNA concentration constant in Optimem serum free media. After 24 hours cells were treated with 10 nM estradiol where indicated. Samples were harvested and analyzed after 24 hours of treatment. Bars represent mean ± SEM of triplicate samples. * Different from ERα alone (bar 5) p<0.05. B. HeLa cells were transiently transfected and treated as in A with the addition of 25 ng of pCR3.1 NCOA expression constructs or control vector. Bars represent mean ± SEM of triplicate samples. * Different from ERα with each respective NCOA alone (bars 5, 13, 21) p<0.05. C. Western blot analysis of ERα expression in transfected cells. Transfected cells were harvested and 100 µg of total protein was analyzed for ERα expression by Western blot. The blot was stripped and probed with an antibody against tubulin to control for loading.

Our data suggest that Ets1 expression increases the sensitivity of MCF7 cells to estradiol signaling. Previous studies have suggested that Ets1 may interact with ERα to stimulate ligand-independent transcriptional activation [26]. Similarly, we transfected HeLa cells with an estrogen responsive reporter (ERE2-TK-LUC) and mammalian expression constructs for human Ets1 and ERα (Figure 4A). The addition of estradiol and Ets1, either alone or in combination, in the absence of ERα, did not alter the activity of ERE2-TK-LUC. Thus, Ets1 alone is unable to activate the ERE2-TK-LUC promoter. In contrast, expression of ERα stimulated ERE2-TK-LUC 17-fold and the addition of exogenous estradiol stimulated reporter activity 50-fold. Ets1, in the presence of ERα, resulted in a 30-fold stimulation of the promoter which was augmented to 60-fold by estradiol. These data are similar to a previous study [26], showing that Ets1 can induce ligand and AF2-independent activation of ERα. Transcriptional activation by ERα alone in the absence of both Ets1 and additional estradiol suggests that despite being transfected in Optimem serum-free media there may be residual estrogens in the cells.

Since the p160 nuclear receptor coactivator family cooperates with both estrogen receptors and Ets transcription factors and is correlated with Ets transcription factor expression in breast cancers [5], [22], we wanted to determine if expression of p160 nuclear receptor coactivators could potentiate Ets1-induced activation of ERE2-TK-LUC (Figure 4B). In the absence of ERα, the addition of NCOAs had no effect on the activity of ERE2-TK-LUC in either the absence or presence of Ets1. In contrast, the addition of NCOA1 increased ERα-dependent activation from 17-fold (Figure 4A) to approximately 100-fold (Figure 4B). Stimulation with estradiol elevated the ER-dependent activity to 176-fold. Inclusion of Ets1 without estradiol stimulated promoter activity to 222-fold, a level slightly higher than that induced by estradiol. Finally, the addition of estradiol along with ERα and Ets1 stimulated a 356-fold response. The expression of NCOA2 gave results similar to those of NCOA1 with fold stimulations for ERα, ERα+estradiol, ERα+Ets1 and ERα+Ets1+estradiol of 107, 218, 182 and 341, respectively. In comparison, NCOA3 induced promoter activity in a pattern similar to NCOA2, but was approximately only one third as stimulatory as NCOA1 or NCOA2. Taken together, these data indicate that p160 nuclear receptor coactivators can cooperate with Ets1 to induce transcription of estrogen-responsive genes in the absence of exogenous estradiol suggesting that they may contribute to the development of ligand-independent activation of ER targets. Finally, we measured the expression of ERα in HeLa cells transfected with ERE2-TK-LUC, pSG5-Ets1, pCDNA-ERα and NCOA1 and treated with and without estradiol (Figure 4C). While transfection of Ets1 and NCOA1 stimulated activity of ERE-TK-LUC, this effect was not due to increased expression of ERα.

### Ets1 Increases the Expression of ERα Target Genes in MCF7 Cells

**Figure 5:**
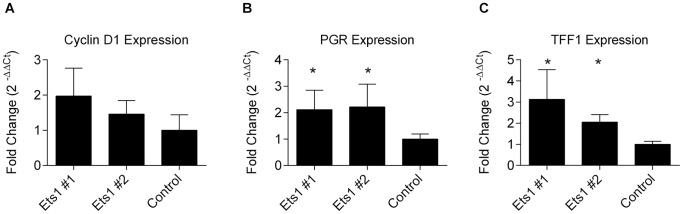
Ets1 increases expression of ERα target genes. Ets1-expressing stable cell lines and MCF7 control cells were analyzed by qRT-PCR for expression of Cyclin D1 (A), PGR (B) and TFF1 (C). * Significantly different than control MCF7 cells by t-test, p<0.05.

To determine if Ets1 could also stimulate expression of ER target genes in the Ets1-expressing MCF7 cells, we measured mRNA transcripts of three established ERα target genes: cyclin D1(*CCND1*), *PGR* and trefoil factor 1 (*TFF1*) [46]. Expression of *PGR* and *TFF1* is significantly increased in the Ets1 clones relative to MCF7 control cells while the increase in expression of *CCND1* does not reach the level of significance (Figure 5), indicating that Ets1 expression in breast cancer cells can stimulate the expression of endogenous estrogen-dependent genes.

### GST-Ets1 can Form Stable Complexes Containing Both ERα and p160 Nuclear Receptor Coactivators

**Figure 6:**
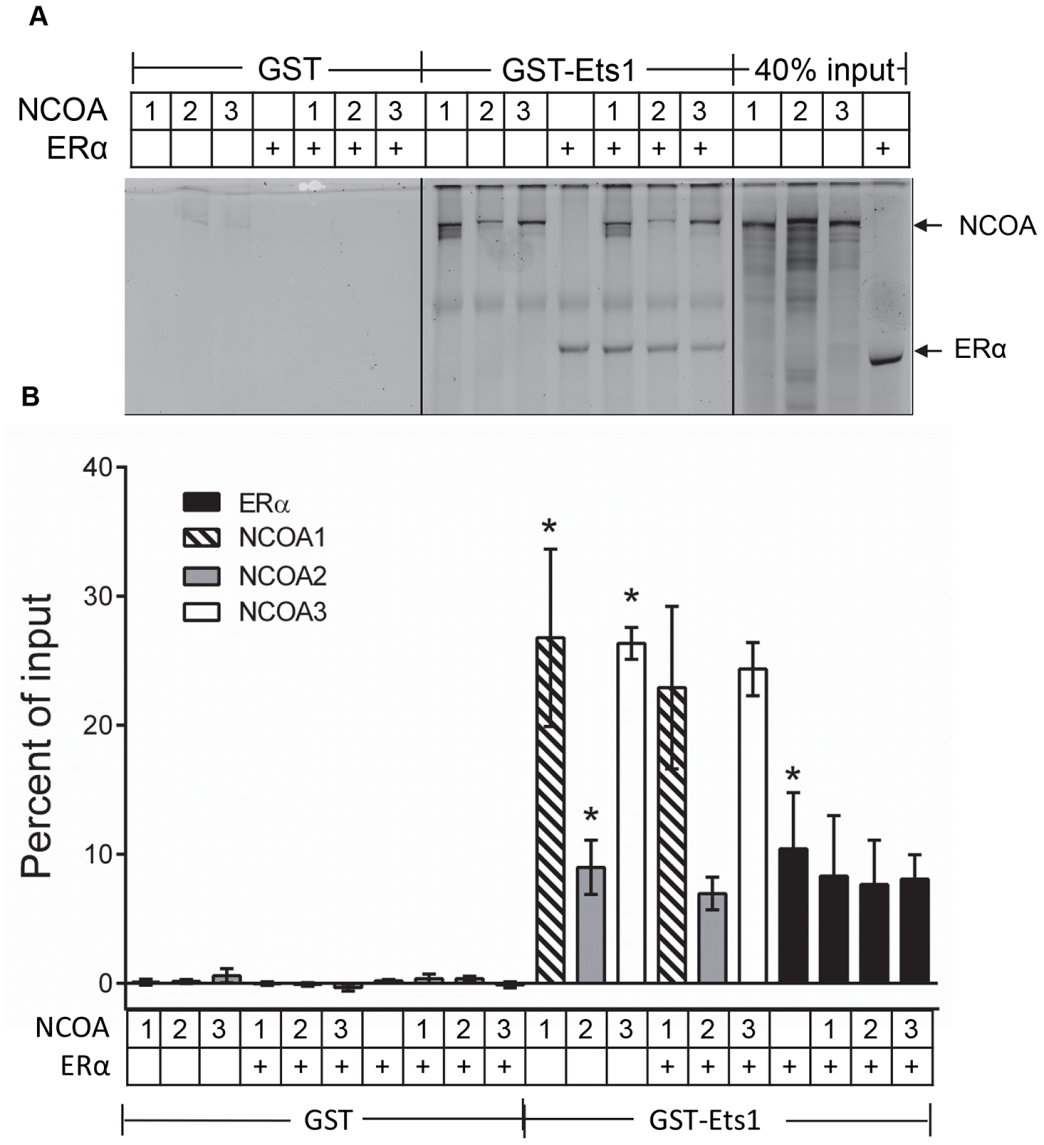
Ets1 can form stable complexes containing both ERα and p160 nuclear receptor coactivators. A. Fluorescently labeled ERα and NCOA1, 2 or 3 were incubated alone or in combination with GST or GST-Ets1 bound to glutathione sepharose. Complexes were separated by SDS-PAGE and bound Green_Lys_-labeled proteins were imaged on a Typhoon 9410**.** Incubation components are listed at image top. Arrows indicate the Green_Lys_ labeled NCOA and ERα proteins. B. The scanned images were quantified by fluorescent imaging analysis. Bars are mean ± SEM expressed as percent of total input bound of three separate experiments. Incubation components are listed at the bottom of the figure. Where combinations are listed twice, the hatched, grey, and white bars indicate respective NCOAs bound and black bars indicate ERα bound. * Significantly different from respective GST control p<0.05 by Fisher’s LSD test. Binding of ERα and NCOAs in combination with GST-Ets1 was not significantly different from binding individually.

The ability of Ets1 and p160 nuclear receptor coactivators (NCOAs) to cooperatively stimulate transcriptional activity of an estrogen responsive reporter suggests that Ets1, ERα and NCOAs may physically interact to form a transcriptional activation complex. To test for this interaction, we measured the binding of fluorescently labeled ERα and NCOA1, 2, and 3 to GST-Ets1 in solution alone and in combination (Figure 6, Figure S3). We found that when incubated separately; purified GST-Ets1 fusion bound 27%, 9%, and 26% of labeled NCOA1, NCOA2 and NCOA3, respectively. GST-Ets1 also bound 10% of labeled ERα input. The GST control did not bind either NCOA proteins or ERα. When incubated in combination, binding of these components was unchanged. This GST-pulldown analysis confirms that Ets1 can physically bind to both ERα and nuclear receptor coactivators and that they do not compete for binding. Thus, a stable complex containing all three proteins can be formed.

## Discussion

We show that Ets1 expression increases cellular proliferation, colony formation, migration and invasion *in vitro* (Figure 2 & Table 1) and increases 17β-estradiol-dependent tumor growth *in vivo* (Figure 3). *In vitro* analyses of transcriptional activation and protein interactions (Figures 4–6) suggest that these effects may be at least partially due to the ability of Ets1 to cooperate with ERα and nuclear receptor coactivators to activate estrogen responsive gene promoters.

Recent data suggest recurrence is greater in patients with elevated tumor expression of Ets factors and members of the p160 nuclear receptor coactivator family [5]. Since Ets factors regulate the expression of the Her2 epidermal growth factor receptor, matrix metalloproteinases and other factors involved in proliferation, invasion and angiogenesis, elevated Ets1 expression may upregulate these genes that facilitate invasion and metastasis. In support of this, we identified increases in colony formation and cellular motility upon Ets1 expression in ERα-positive human breast cancer cells (Table 1 and Figure 2). Three of the four cell lines tested exhibited increased colony formation in response to Ets1 expression; however, the BT-474 cell line, the only one of these ERα positive, luminal breast cancer lines that expresses ErbB2 [41], did not. Further, while colony formation was slightly increased by the inclusion of estradiol, this increase was not statistically significant (Figure 2C). The relative estradiol insensitivity of the clonogenic studies may be due to the rich cell culture conditions including: 25 mM glucose, 4 mM glutamine and 10% FBS. In contrast, MCF7 tumor growth in ovariectomized nude mice with and without Ets1 was strictly dependent upon estradiol (Figure 3A). Interestingly, MCF7 cells injected in cellulose pellet implanted mice were viable throughout the study, producing a bioluminescent signal despite the lack of tumor growth (Figure 3C). While previous studies have suggested that the loss of estradiol signaling induces apoptotic cell death in MCF7 cells [47], these luminescence data suggest that some cells are resistant to or protected from these apoptotic mechanisms. This protective mechanism may be due to interactions of the MCF7 cells with the 50% Matrigel solution used for cell injection, as it contains both extracellular matrix components and growth factors to support tumor propagation [48], [49].

Conversely, the Ets1-expressing tumors grown in the presence of 17β-estradiol were larger in size than the MCF7 control tumors (Figure 3A) with increased central areas of necrosis (Figure 3D). The Ets1-expressing tumors also exhibited elevated TUNEL staining which may correlate to the increased necrosis (Figure 3F), but did not exhibit elevated Ki67 staining despite their increased growth rate and size (Figure 3E). Ki67 protein is present during all active phases of the cell cycle (G_1_-S-G_2_-M), but is absent from resting cells in G_0_, making it a useful marker to determine the growth fraction of a tumor and a useful prognostic marker in breast cancer [50]. Unfortunately, Ki67 staining does not relate to the time required for completion of an intermitotic cycle. In addition, Ki67 staining was only conducted at the end of the study in tumors dissected from the mammary fat pad. Dissection may have removed the leading edge of the tumor which may have exhibited a higher proliferative rate [51]. Additionally, tumor growth may have slowed by this point, particularly in large tumors with necrotic centers suggesting these tumors outgrew their blood supply. Interestingly, our findings are not unique [52], [53]. In a recently published paper exploring the role of the hypoxia-inducible gene carbonic anhydrase IX (CA9) in tumor resistance to bevacizumab, the authors discovered that tumors overexpressing CA9 exhibited increased cellular proliferation in culture and increased tumor growth *in vivo.* Similar to our study, the larger CA9-expressing tumors exhibited increased necrosis and increased apoptosis, with no elevation of Ki67 staining in the larger tumors [53]. In this study, the authors hypothesize that the necrosis may represent regions in which proliferation was elevated despite hypoxia and substrate depletion by CA9 maintenance of an alkaline intracellular pH. A similar phenomenon may be occurring in our Ets1 expressing tumors since CA9 has been proposed as an Ets1 regulated gene [54].

Despite the association of Ets transcription factors with angiogenesis, we did not identify any increases in proliferating endothelial cells based on dual KI67/CD31 staining in the Ets1 expressing tumors (Figure S2). Thus, increases in breast cancer angiogenesis associated with Ets1 are likely due to elevated Ets1 expression in endothelial cells [55].

Elevated expression of both Ets1 and Ets2 has been identified in invasive breast cancers and correlated with increased expression of p160 nuclear receptor coactivators [5]. In addition, siRNA-mediated knockdown of p160 nuclear receptor coactivators in MCF7 cells results in altered cell proliferation, apoptosis and expression of ERα target genes [56]. Further, Ets1 has been shown to interact with ERα to induce ligand independent transcriptional activation [26]. Ets factors are both upstream mediators inducing ErbB2/Her2/neu expression [57] and downstream targets of growth factor signaling through the Ras/Raf/MAPK pathway which has been associated with tamoxifen resistance [58] suggesting that Ets1 expression may play a role in or lead to estrogen- independent growth. In the current study, we find that in addition to increasing the rate of cellular proliferation, the expression of Ets1 also increased the growth rate of MCF7 tumors in an orthotopic, nude mouse model (Figure 3), but only in an estrogen-dependent manner. The interdependence of typical genomic estrogen receptor activation and growth factor signaling pathways has been illustrated by studies in which inhibition of either growth factor receptors or estrogen receptors also suppresses the corresponding pathway [59]. In a similar manner, the expression of Ets1 as a target of the MAP kinase pathway may augment estradiol signaling. Although Ets1, acting alone, may activate genes to stimulate cellular proliferation, the continued estradiol-dependence of tumor growth suggests that the cooperative interaction between Ets1 and ERα is responsible. We validated cooperative activation by Ets1 and ERα of a luciferase reporter containing two estrogen response elements and further found that cotransfection of NCOAs augmented this Ets1 stimulated transcriptional activity (Figure 4). We also determined that the Ets1 expressing MCF7 clones had increased expression of endogenous ERα target genes, *PGR* and *TFF1*, compared to control MCF7 cells (Figure 5). In this regard, our data indicate that Ets1 may hypersensitize estrogen-responsive promoters to estrogen, similar to the increased sensitivity exhibited by some models of endocrine-resistant breast cancer [7].

Interestingly, while ERα targets with full or partial estrogen response elements were significantly stimulated by Ets1 expression, cyclin D1, an ERα target activated through non-canonical ERα binding [60] was not. This may suggest that Ets1 differentially activates ERα gene targets depending on the specific conformation of coactivator complexes. Ets1 binding to the A/B/C domains of the nuclear hormone receptors [26] may bring Ets1-bound NCOAs to the ERα transcriptional activation complex. In fact, we found that both ERα and NCOAs were able to bind GST-Ets1 in a non-competitive manner suggesting that stable complexes containing all three proteins were formed in GST-pulldown analysis (Figure 6).

Thus, binding of Ets1 to nuclear hormone receptors may serve as a bridging molecule between the receptors and nuclear receptor coactivators to activate receptors in a ligand-independent manner or sensitize nuclear hormone receptors to low ligand levels. Further, the increased sensitivity to estradiol with the addition of Ets1 in tumor growth response, cellular responses, and transcriptional activation suggests that the complexes formed will continue to allow nuclear hormone receptor binding of ligand to stimulate NCOA binding through the AF2 domain of ERα. In this regard, Ets transcription factors may serve as a molecular switch that increases the sensitivity of breast cancers to estrogen-stimulated growth by recruiting NCOA coactivators to estrogen responsive genes. Ultimately, this action may lead to hormone-independence and resistance to hormone based therapies. Thus, Ets1 may represent an additional target for the prevention and treatment of hormone-independent breast cancer.

## Supporting Information

Figure S1 **Apoptosis induced by serum starvation.** MCF-7 control and Ets1 #1 cells were serum starved for 24 hours then stained with annexin V and analyzed by flow cytometry. Bars represent mean ± SEM.
(PDF)

Figure S2 **Proliferating blood vascular endothelial cells in tumors were measured by dual Ki-67 and CD31 staining.** Four fields per tumor (n = 7) were stained, counted and averaged. Bars represent mean ± SEM of average counts in each tumor group.
(PDF)

Figure S3 **Fluorescently labeled and Gelcode blue stained images from** **Figure 6** **.** A. Fluorescently labeled ERα and NCOA1, 2 or 3 were incubated alone or in combination with GST or GST-Ets1. Complexes were separated by SDS-PAGE and Bound GreenLys labeled proteins were imaged on a Typhoon 9410. B. Following analysis of fluorescently stained proteins, gels were stained with Gelcode Blue and imaged on a Biorad ChemiDoc XR.
(PDF)

Table S1 **Primer sequences and amplicon sizes for selected genes.**
(PDF)

Table S2 **Growth inhibition of Ets1-expressing MCF-7 cells by 4- hydroxytamoxifen.**
(PDF)
