# Supplementary material for: Transcription Factor Ets1 Cooperates with Estrogen Receptor α to Stimulate Estradiol-Dependent Growth in Breast Cancer Cells and Tumors
Source: PLoS One. 2013 Jul 9;8(7):e68815. doi: 10.1371/journal.pone.0068815 (PMC3706316; doi:10.1371/journal.pone.0068815)
Supplement: Figure S1 — Apoptosis induced by serum starvation. MCF-7 control and Ets1 #1 cells were serum starved for 24 hours then stained with annexin V and analyzed by flow cytometry. Bars represent mean ± SEM. (PDF) [file pone.0068815.s001.pdf]

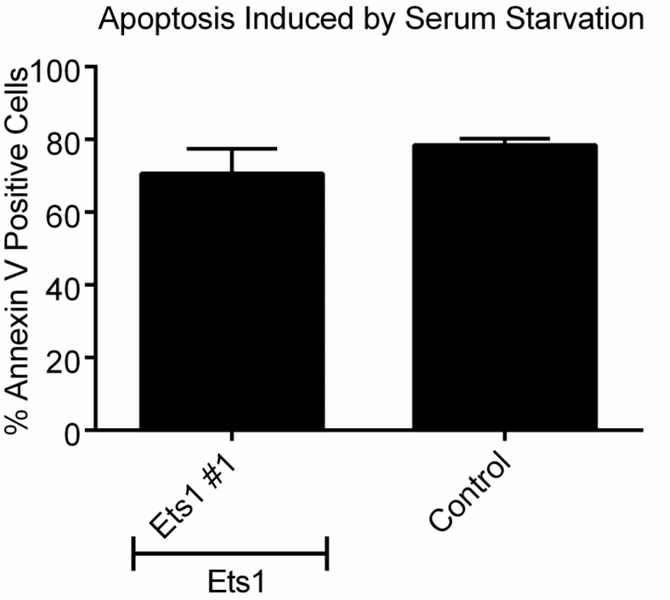

Figure S1. Apoptosis induced by serum starvation. MCF-7 control and Ets1 #1 cells were serum starved for 24 hours then stained with annexin V and analyzed by flow cytometry. Bars represent mean ± SEM.
