# Supplementary material for: Transcription Factor Ets1 Cooperates with Estrogen Receptor α to Stimulate Estradiol-Dependent Growth in Breast Cancer Cells and Tumors
Source: PLoS One. 2013 Jul 9;8(7):e68815. doi: 10.1371/journal.pone.0068815 (PMC3706316; doi:10.1371/journal.pone.0068815)
Supplement: Figure S2 — Proliferating blood vascular endothelial cells in tumors were measured by dual Ki-67 and CD31 staining. Four fields per tumor (n = 7) were stained, counted and averaged. Bars represent mean ± SEM of average counts in each tumor group. (PDF) [file pone.0068815.s002.pdf]

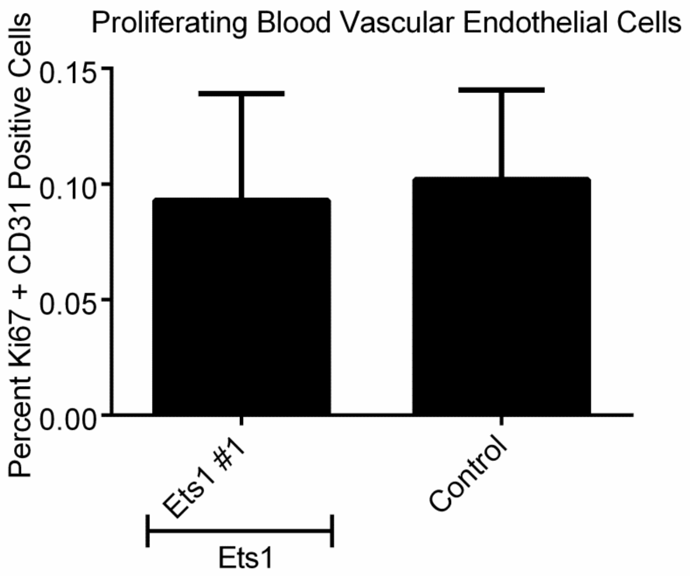

Figure S2. Proliferating blood vascular endothelial cells in tumors were measured by dual Ki-67 and CD31 staining. Four fields per tumor (n=7) were stained, counted and averaged. Bars represent mean ± SEM of average counts in each tumor group.
