# Supplementary material for: Transcription Factor Ets1 Cooperates with Estrogen Receptor α to Stimulate Estradiol-Dependent Growth in Breast Cancer Cells and Tumors
Source: PLoS One. 2013 Jul 9;8(7):e68815. doi: 10.1371/journal.pone.0068815 (PMC3706316; doi:10.1371/journal.pone.0068815)
Supplement: Figure S3 — Fluorescently labeled and Gelcode blue stained images from Figure 6 . A. Fluorescently labeled ERα and NCOA1, 2 or 3 were incubated alone or in combination with GST or GST-Ets1. Complexes were separated by SDS-PAGE and Bound GreenLys labeled proteins were imaged on a Typhoon 9410. B. Following analysis of fluorescently stained proteins, gels were stained with Gelcode Blue and imaged on a Biorad ChemiDoc XR. (PDF) [file pone.0068815.s003.pdf]

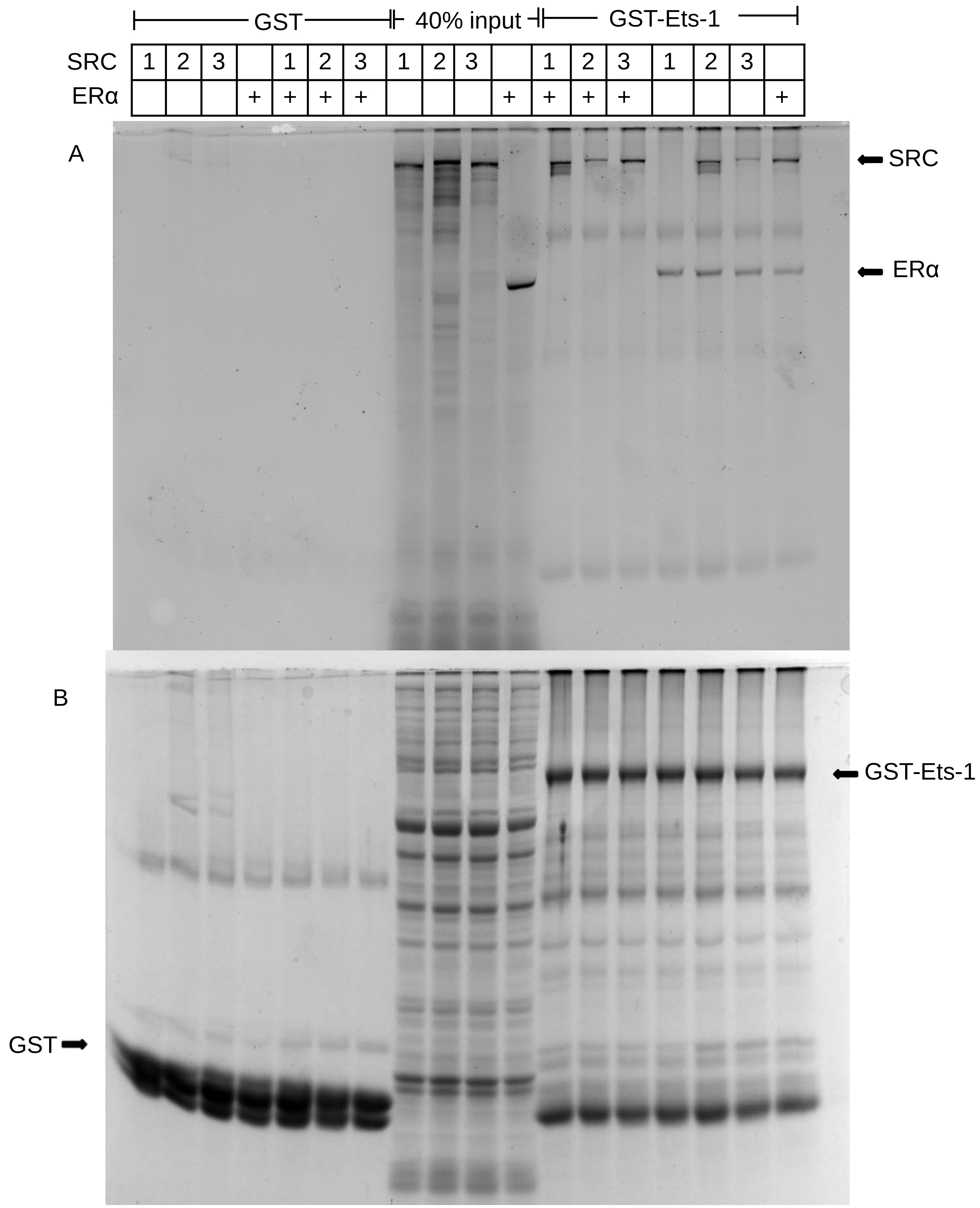

Figure S3. Fluorescently labeled and Gelcode blue stained images from Figure 6. A. Fluorescently labeled ERα and NCOA1, 2 or 3 were incubated alone or in combination with GST or GST-Ets1. Complexes were separated by SDS-PAGE and Bound Green$_{Lys}$-labeled proteins were imaged on a Typhoon 9410**.** B. Following analysis of fluorescently-stained proteins, gels were stained with Gelcode Blue and imaged on a Biorad ChemiDoc XR.
