# Supplementary material for: Transcription Factor Ets1 Cooperates with Estrogen Receptor α to Stimulate Estradiol-Dependent Growth in Breast Cancer Cells and Tumors
Source: PLoS One. 2013 Jul 9;8(7):e68815. doi: 10.1371/journal.pone.0068815 (PMC3706316; doi:10.1371/journal.pone.0068815)
Supplement: Table S1 — Primer sequences and amplicon sizes for selected genes. (PDF) [file pone.0068815.s004.pdf]

Table S1. Primer sequences and amplicon sizes for selected genes

Primer	Sequence (5' to 3')	Size of Amplicon
Cyclin D1 Forward	CCG TCC ATG CGG AAG ATC	86 base pairs
Cyclin D1 Reverse	ATG GCC AGC GGG AAG	
Ets1 Forward	CCG TAC GTC CCC CAC TCC T	130 base pairs
Ets1 Reverse	TGG GAC ATC TGC ACA TTC CA	
HPRT1 Forward	TGC TCG AGA TGT GAT GAA GG	192 base pairs
HPRT1 Reverse	TCC CCT GTT GAC TGG TCA TT	
PGR Forward	TGC CTT ACC ATG TGG CAG ATC CC	130 base pairs
PGR Reverse	ACT GGG TTT GAC TTC GTA GCC CT	
TFF1 Forward	CAT CGA CGT CCC TCC AGA AGA G	105 base pairs
TFF1 Reverse	CTC TGG GAC TAA TCA CCG TGC TG	
