# Supplementary material for: Transcription Factor Ets1 Cooperates with Estrogen Receptor α to Stimulate Estradiol-Dependent Growth in Breast Cancer Cells and Tumors
Source: PLoS One. 2013 Jul 9;8(7):e68815. doi: 10.1371/journal.pone.0068815 (PMC3706316; doi:10.1371/journal.pone.0068815)
Supplement: Table S2 — Growth inhibition of Ets1-expressing MCF-7 cells by 4- hydroxytamoxifen. (PDF) [file pone.0068815.s005.pdf]

Table S2. Growth inhibition of Ets1-expressing MCF-7 cells by 4-hydroxytamoxifen

**Cell Line**	**IC_{50} (µM, 95% confidence interval)**
MCF-7 + empty vector	5.262 – 8.888
Ets1 #1	5.764 – 6.846
Ets1 #2	6.373 – 7.541
